# Resolved-GSERF: A Proficient NMR Approach for Identifying Accurate *J*-Coupling Constants of Targeted Peaks from Spectral Congestion

**DOI:** 10.3390/molecules31132386

**Published:** 2026-07-07

**Authors:** Xiaoqing Lin

**Affiliations:** School of Electronic Information, Zhangzhou Institute of Technology, Zhangzhou 363000, China; linxiaoqing@t.fjzzit.edu.cn

**Keywords:** NMR spectroscopy, *J*-coupling constant, spectral congestion, selective refocusing

## Abstract

SElective ReFocusing (SERF)-based experiments present an effective tool for discerning proton–proton *J*-coupling constants of targeted spectral peaks. Nevertheless, the performance of SERF experiments highly relies on the selective excitation of isolated signals. The extraction of targeted peaks from spectral congestion, even from spectral overlap, generally poses a challenge to SERF-based experiments for accurate analysis of associated coupling constants due to the existence of redundant axial peaks and unwanted couplings. Herein, we propose an NMR method called resolved-GSERF to address this challenge and achieve accurate *J*-coupling measurements on targeted spectral peaks. In this approach, the selective magnetization transfer scheme with an appropriate spin-lock mixing time is employed to isolate the targeted peak signal from the spectral congestion region, thereby enhancing spectral sensitivity and resolution. The slice-selection-based GSERF refocusing encoding scheme is subsequently applied to extract *J*-coupling constant information of the targeted peak. The experimental results on complex chemical and biomedical samples suggest that the proposed approach offers a practical solution to the challenge of spectral congestion for identifying accurate *J*-couplings.

## 1. Introduction

Benefitting from non-invasive detection and efficient analysis of composition and molecular structures, nuclear magnetic resonance (NMR) spectroscopy finds extensive applications in diverse fields, such as biochemistry [1,2,3], petrochemistry [4,5,6], and medicine [7,8,9]. The elucidation of NMR spectra facilitates the acquisition of *J*-couplings, which provide valuable information related to molecular structure elucidation and composition analysis. In general, *J*-coupling constants reveal information regarding internuclear relations, configuration, conformation, and substitution positions. In one-dimensional (1D) ^1^H spectra, all *J*-coupling information of protons is presented in the form of coupling splitting and gives multiplet peaks in the resulting spectra. For some small molecules, the distribution of multiplet peaks on 1D ^1^H spectra is relatively dispersed, and it would yield well-resolved peaks in the 1D NMR spectra. Then, molecular structures can be reconstructed by analyzing peak splitting patterns of multiplet peaks and measuring corresponding *J*-coupling constants. However, for complex molecular systems, abundant multiplet peaks with extensive *J*-coupling splitting are present in the acquired 1D ^1^H spectra with a limited range of chemical shift dispersion. This inevitably leads to spectral congestion with peak crowding or even overlapping, which makes peak assignments and their *J*-coupling determination for spectral analysis challenging. The challenge of spectral congestion can be mitigated by extending 1D NMR to two-dimensional (2D) and multi-dimensional NMR and dispersing spectral information in separate dimensions. For instance, 2D *J*-resolved spectroscopy [10,11] enhances spectral resolution by separating chemical shifts and *J*-couplings along two different dimensions. Another approach to alleviating peak congestion is TOtal Correlation SpectroscopY (TOCSY) [12], which employs a spin-lock module to sufficiently diffuse spin interactions and produce correlated magnetization during the mixing period. Designed to detect coupling correlations among protons in molecules, it provides information regarding protons coupled to a given proton, including three-bond couplings and long-range couplings. An improved derivative of the traditional TOCSY technique, selective TOCSY [13,14], is meticulously proposed to target a given proton and extract its coupling correlation network. In the resulting 1D selective TOCSY spectrum, only the peaks coupled to the given proton would appear. Benefiting from this characteristic, this innovative approach can provide an effective filtering method for extracting targeted peaks from the spectral congestion of complex samples with enhanced spectral resolution.

However, for protons that are intricately coupled with multiple protons, spectral peaks derived from the aforementioned methodologies exhibit multiple splitting patterns, rendering the analysis process of their coupling constants complex. Moreover, in practical applications, the 1D ^1^H NMR spectrum often presents challenges in distinguishing *J*-coupling splitting patterns because of low spectral resolution, making accurate *J*-coupling measurement even more difficult. The low spectral resolution primarily stems from numerous *J*-coupling splittings within the limited chemical shift range in the 1D ^1^H spectrum. Additional factors contributing to low spectral resolution include slight non-uniformity of magnetic fields. In such cases, it becomes challenging to observe small *J*-coupling splitting, leading to misinterpretation of spectral splitting, thereby affecting the correct determination of molecular structure. Furthermore, changes in solute concentration can have a significant impact on the shape of peaks in a spectrum. This is often due to alterations in the chemical environment and interactions between molecules, which may result in broadening of spectral peaks. SElective ReFocusing (SERF) technique [15,16], a modified variant of conventional 2D *J*-resolved spectroscopy, is proposed based on the excitation of passive spins succeeded by the doubly selective echo module. In the doubly selective echo module, both chosen protons, i.e., identical passive protons and other active ones, are evolved under selective inversion during the variable echo period *t*_1_. This results in a distinctive *J*-resolved spectrum that solely reveals *J*-couplings between active and passive protons. However, since the initial SERF experiments are only capable of extracting *J*-coupling information for a pair of protons in a single experiment, the measurements of full *J*-couplings for a targeted proton require multiple experiments with prolonged experimental time. To enhance experimental efficiency, the spatial frequency encoding technique known as the slice selection technique [17] is incorporated to correlate NMR frequency with spatial position, selectively exciting different signals in various slices. This innovative approach, called GSERF [18], allows the acquisition of full *J*-coupling constant information for a targeted proton in one experiment, additionally revealing its coupling correlation network. Recently, several improved variants of GSERF have been proposed to enhance its performance. These proposed GSERF-based protocols include enhancing experimental efficiency [19], incorporating broadband homonuclear decoupling [20,21] with pure shift presentation [22], accelerating experimental acquisition by utilizing real-time homonuclear decoupling techniques [23,24], determining *J*-couplings between equivalent protons in a symmetric molecule [25], and recovering high-resolution *J*-coupling information from inhomogeneous magnetic fields [26].

GSERF-based methods described above are primarily applied to the case of isolated peaks in the 1D ^1^H spectrum. However, these methods generally suffer from limitations when it comes to spectral congestion cases with crowding and overlapping peaks. Improved GSERF-based experiments are further proposed for high-resolution *J*-coupling measurement on congested spectral peaks. One specific experiment, PSYCHEDELIC [27], which utilizes small flip-angle swept-frequency pulses and weak gradients to achieve selective spin inversion [28], aims to overcome the challenges of measuring *J*-coupling constants in crowded peaks by employing a 3D method for improving spectral quality. However, this PSYCHE-based GSERF approach sacrifices spectral sensitivity and experimental efficiency, and it retains redundant axial peaks that can interfere with the accurate measurements of the targeted peak. In addition, the slice-selection-based GSERF approaches, which use selective pulses and weak gradients to achieve spatial frequency encoding, are proposed by using selective COSY strategies to extract targeted peaks in congested regions [29,30]. However, the utilization of selective COSY leads to low signal sensitivity in these slice-selection-based GSERF methods. Fredi et al. developed TOCSY-GSERF [31] and Kakita et al. subsequently proposed G-SERF-PSYCHE-TOCSY [32], both of which adopt PSYCHE as the core *J*-refocusing module built upon spin population selection principles. In Kakita’s G-SERF-PSYCHE-TOCSY configuration, both the direct and indirect dimensions contain chemical shift information, and all related signals align along the spectral diagonal. Full sampling over the indirect dimension is essential to collect complete *J*-coupling information of a target proton, leading to lengthy experimental durations. Although diagonal arrangement partially mitigates axial peak interference, residual axial signals still hinder spectral analysis.

PSYCHE [28,33] and slice-selection [20] are two classic *J*-refocusing techniques with different working mechanisms and respective merits. As previously documented in published studies, PSYCHE acquires signals across all sample slices to guarantee baseline sensitivity, whereas slice-selection possesses several unique superiorities that PSYCHE cannot achieve [26,34,35]: As validated in multi-slice spatially encoded NMR experiments, spatially segregated slice signals facilitate the integration of multiband pulses and Fourier phase encoding to boost signal-to-noise ratio (SNR); slice-selection frameworks deliver intrinsic robustness against magnetic field inhomogeneity; slice-selection pulse schemes maintain full compatibility with real-time sampling techniques to reduce total experimental acquisition duration. Given these literature-verified merits of slice-selection, combining selective magnetization transfer with slice-selection-based GSERF has great potential to circumvent the deficiencies of existing PSYCHE-based approaches and boost experimental performance.

In this study, we propose an efficient GSERF approach, called resolved-GSERF, to achieve accurate *J*-coupling determination from the spectral congestion case, along with the practical consideration of signal intensity and experimental efficiency. Our core goal is to offer a practical solution for crowded spectra rather than outperforming all established NMR techniques comprehensively. The proposed resolved-GSERF adopts the concept of targeted signal separation by magnetization transfer and accurate *J*-coupling determination by the slice-selection-based GSERF. Appropriate spin-lock mixing time is used in the selective magnetization scheme for targeted screening of the desired signals of a given proton, and the slice-selection-based GSERF refocusing encoding scheme is subsequently applied to identify corresponding *J*-coupling information of the given proton. Benefiting from the use of the selective magnetization transfer scheme, the resolved-GSERF gets rid of the influence of uninformative axial peaks and unwanted couplings, providing satisfactory resolution and high sensitivity. To show the ability of the resolved-GSERF to determine unambiguous *J*-coupling and multiplet structure analysis on complex samples, the resolved-GSERF experiments are performed on different practical samples. The experimental results on complex chemical and biomedical samples suggest that the proposed approach offers a practical solution to the challenge of spectral congestion, particularly peak overlapping.

## 2. Results

**Figure 1:**
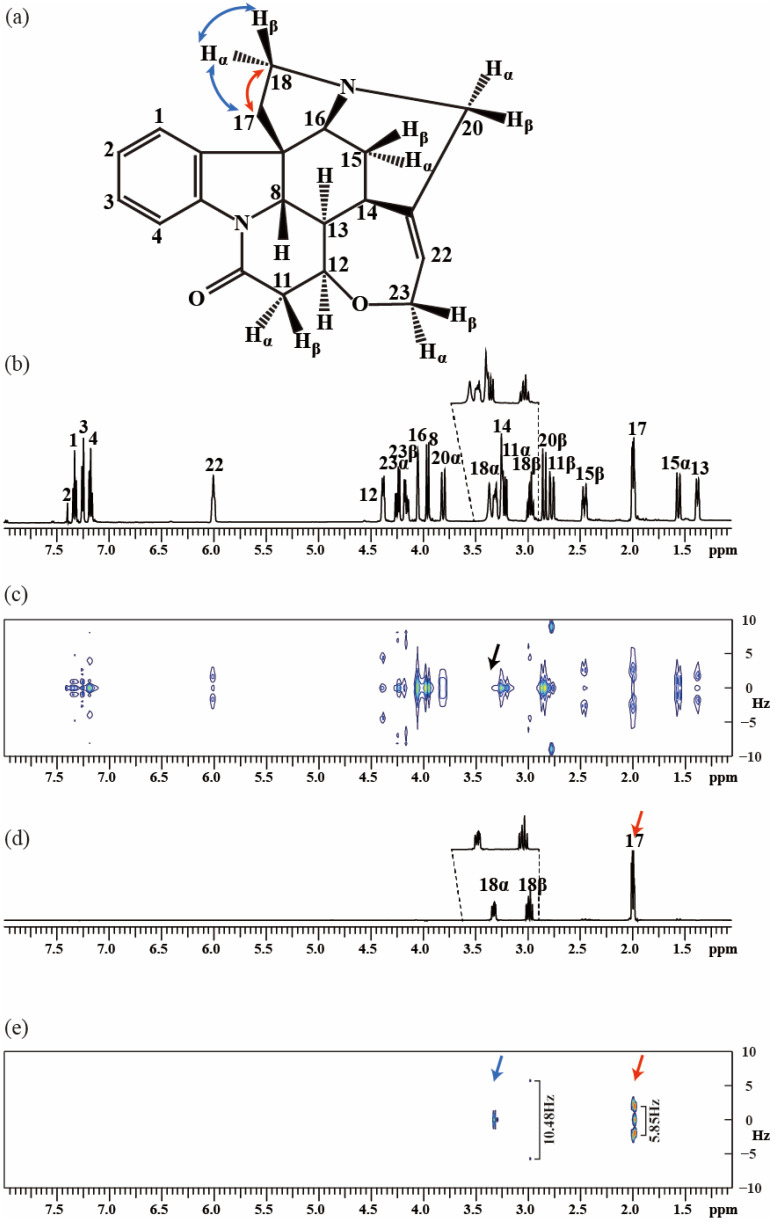
Experimental results for a solution sample of strychnine: (**a**) Molecular structure of strychnine, where the red and blue arrows represent the coupling correlation networks of protons H_17_ and H_18*α*_, respectively. (**b**) 1D ^1^H NMR spectrum with assigned peaks. (**c**) Conventional GSERF experiments for proton H_18*α*_. The black arrow indicates the frequency center of the selective pulse in GSERF. (**d**) 1D selective TOCSY spectrum obtained by the resolved-GSERF on proton H_17_ with a short spin-lock mixing time of 50 ms. (**e**) 2D resolved-GSERF spectrum for proton H_18*α*_. The red and blue arrows indicate the selected proton H_17_ and proton H_18*α*_ in selective TOCSY and GSERF schemes, respectively.

To show the implementation details of the resolved-GSERF, we perform experiments on an aqueous solution of 200 mM strychnine (Figure 1). Strychnine is an alkaloid extracted from the Strychnos nux-vomica tree, and it can be clinically used in the treatment of paresis or amblyopia [36]. As one of the complex natural products, strychnine contains seven carbon rings and six chiral centers, shown as the molecular structure in Figure 1a. Because of its relatively complex molecular structure and limited chemical shift range, the 1D ^1^H NMR spectrum generally suffers from spectral congestion [Figure 1b], e.g., peak crowding observed in the spectral region between 3.10 ppm and 3.50 ppm. This congestion would pose a challenge in peak assignments and *J*-coupling measurements. Herein, we take proton H_18*α*_ as an example to illustrate its ability to accurately measure the *J*-coupling of targeted peaks from the crowded spectral regions. Intuitively, we can use selective pulses to excite proton H_18*α*_ and extract its *J*-coupling information from the acquired 1D NMR spectrum. However, the chemical shift difference between the desired proton H_18*α*_ and adjacent protons, e.g., proton H_14_, is relatively slight, which makes it difficult to directly excite proton H_18*α*_ by using the selective pulses. In the conventional GSERF experiment (see Figure S1 in Supplementary Materials for the pulse sequence), the selectivity of the selective pulse in the slice-selection module depends on the minimum chemical shift difference between protons in the entire coupling system. However, the signal sensitivity in the slice-selective scheme depends on the bandwidth of the selective pulses. The enhanced selectivity of the selective pulse generally leads to reduced signal sensitivity. To maintain signal sensitivity, the selectivity of the selective pulse is typically determined by the minimum chemical shift difference between the protons in the *J*-coupling network of the targeted proton. This approach may introduce unnecessary incoherent *J*-coupling networks and interfere with the experimental analysis in the conventional GSERF results. For instance, in the conventional GSERF experiments on strychnine samples [see Figure 1c], the selectivity of the selective pulses depends on the minimum chemical shift difference between the protons in the coupling network of the targeted proton H_18*α*_. Because of strong coupling effects, the *J*-coupling of protons H_23*α*_ and H_23*β*_ cannot be refocused during the *t_1_* period. Consequently, the resulting spectrum [Figure 1c] shows a spurious *J*-coupling correlation signal between protons H_23_ and H_18*α*_. However, the molecular structure analysis suggests that there is no *J*-coupling correlation between protons H_23_ and H_18*α*_. Additionally, due to the insufficient selectivity of the selective pulses covering protons H_14_ and H_11*α*_, the coupling networks of these protons and the associated *J*-coupling splittings are also given in the resulting 2D GSERF spectrum. In the resolved-GSERF experiment, we selectively excite a resolved peak of proton H_17_ and avoid direct excitation on the peak of proton H_18*α*_ in the crowded spectral region. Then the spin-lock mixing time of the DIPSI2 is properly set as a short value of 50 ms to transfer the direct coupling correlation from proton H_17_ to protons H_18*α*_ and H_18*β*_, and a 1D selective TOCSY spectrum showing peaks from proton H_17_ and its directly coupled protons H_18*α*_ and H_18*β*_ are obtained in Figure 1d. At this short mixing time of 50 ms, only ^3^*J*-mediated magnetization transfer is efficient enough to yield detectable peaks, while long-range ^4^*J* couplings are too weak to produce observable resonances. Extension of spin-lock mixing time enhances magnetization transfer efficiency for ^4^*J* couplings to realize their detection, accompanied by extra resonance peaks derived from non-target remote spin couplings. To further recover accurate *J*-coupling information of proton H_18*α*_, the slice-selection-based GSERF refocusing encoding module of the resolved-GSERF is used to selectively excite the peak of proton H_18*α*_ and yield the desired 2D resolved-GSERF spectrum [Figure 1e]. In the resolved-GSERF experiment, the bandwidth of the selective pulse in the selective magnetization transfer scheme depends on the chemical shift difference between the given peak and its adjacent peak. Because the selected proton H_17_ is an isolated peak, the selective bandwidth can be set relatively large. The bandwidth of the selective pulse in the slice-selection-based GSERF refocusing encoding module of resolved-GSERF depends on the minimum chemical shift difference between the coupled protons. In this 2D spectrum, the peak of proton H_18*α*_ is shown as a singlet, and its directly coupled peaks from protons H_18*β*_ and H_17_ are shown as a doublet. Accurate *J*-coupling constants can be obtained by measuring these two doublet peaks along the indirect *F*1 dimension. For example, the *J*-coupling constant between H_18*α*_ and H_18*β*_ is 10.48 Hz, and it is 5.85 Hz between H_18*α*_ and H_17_. The experimental results on strychnine demonstrate that the resolved-SERF holds the ability to restore accurate *J*-coupling information of peaks from the crowded spectral region. Moreover, the resolved-GSERF can yield a clean spectrum and eliminate the adverse effects of irrelevant coupling spin systems, even when the same selective pulse is used in the GSERF module of conventional GSERF and resolved-GSERF. Therefore, resolved-GSERF holds promise for the analysis of complex samples by providing accurate molecular structure information. To validate the accuracy of measured *J*-coupling constants, we also perform comparison experiments on a solution sample of *γ*-aminobutyric acid by using the resolved-GSERF and conventional GSERF methods, and the relevant results are given in the Supplementary Materials.

**Figure 2:**
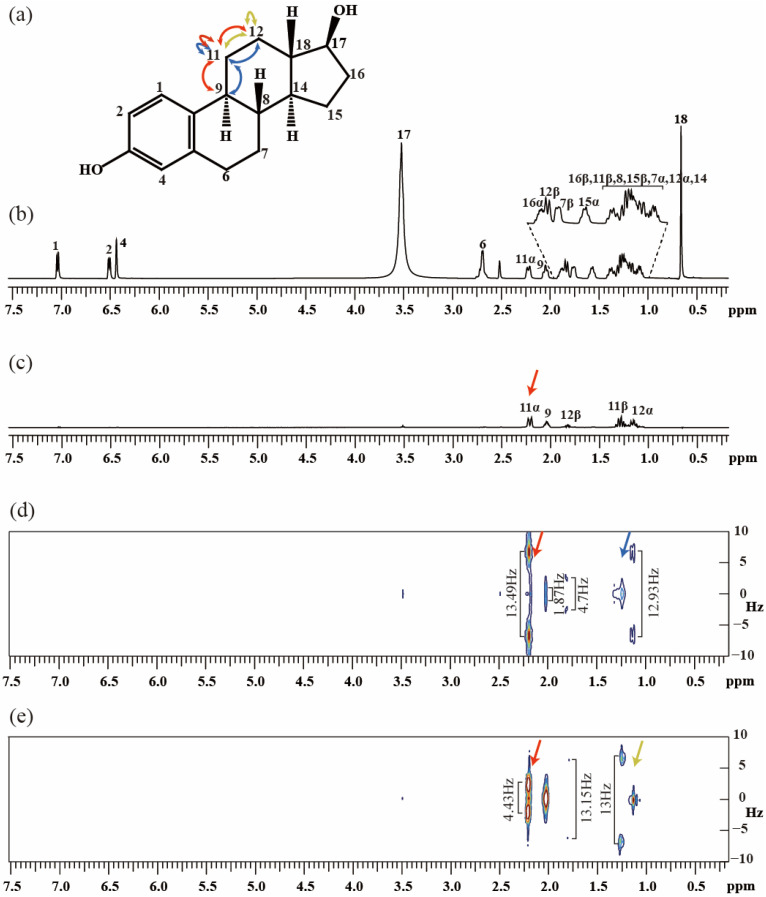
Resolved-GSERF experiments on a challenging sample of 17*β*-estradiol: (**a**) The molecular structure of 17*β*-estradiol, along with identified coupling correlation networks of protons H_11*α*_, H_11*β*_, and H_12*α*_ marked by red, blue, and yellow arrows, respectively. (**b**) 1D ^1^H NMR spectrum with assigned protons. (**c**) 1D selective TOCSY spectrum by the resolved-GSERF on proton H_11*α*_ with a short spin-lock mixing time of 30 ms. (**d**,**e**) 2D resolved-GSERF spectra for protons H_11*β*_ and H_12*α*_, respectively. The red arrow indicates the selected proton H_11*α*_ in the selective TOCSY scheme; blue and yellow arrows indicate the targeted protons H_11*β*_ and H_12*α*_, respectively, in the GSERF scheme.

**Figure 3:**
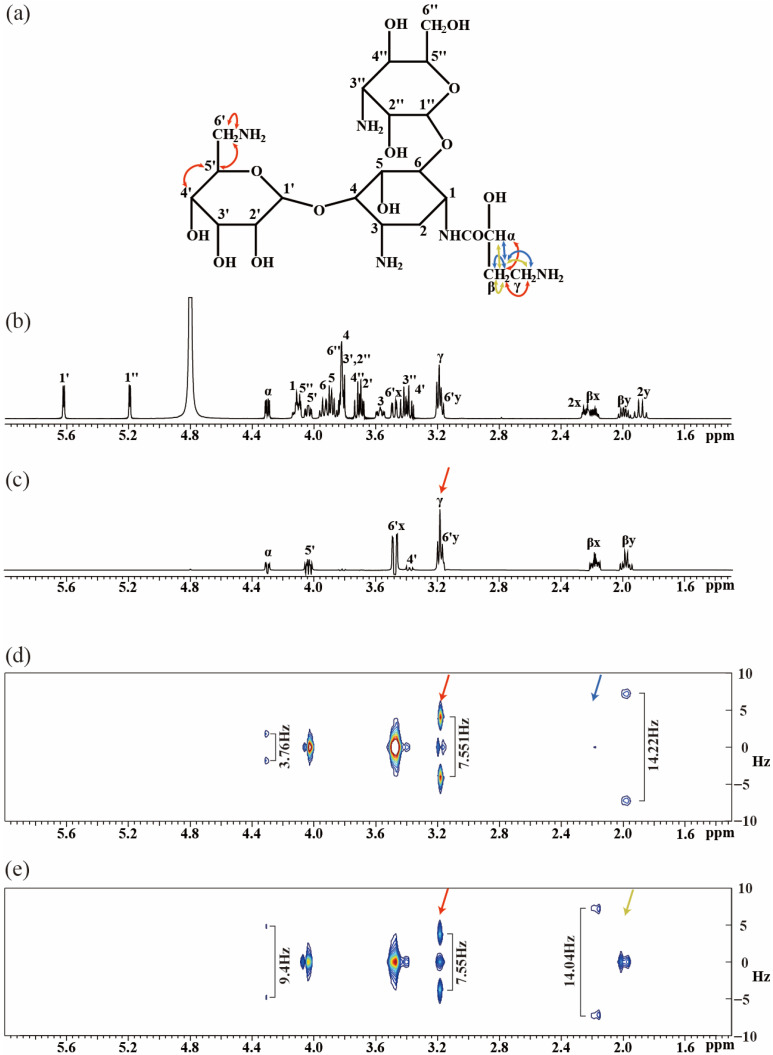
Resolved-GSERF experiments on a medical sample of amikacin: (**a**) Molecular structure of amikacin, along with identified coupling correlation networks of protons H*_βx_*, H*_βy_*, and H*_γ_*, respectively. (**b**) 1D ^1^H NMR spectrum along with assigned peaks. (**c**) 1D selective TOCSY spectrum by the resolved-GSERF for the selective excitation on peaks of protons H*_γ_* and H_6′*y*_ with a spin-lock mixing time of 30 ms. 2D resolved-GSERF spectra for protons (**d**) H*_βx_* and (**e**) H*_βy_*. The red arrow indicates the selected proton H*_γ_* in the selective TOCSY scheme, blue and yellow arrows indicate the targeted protons H*_βx_* and H*_βy_*, respectively, in the GSERF scheme.

To further illustrate the performance of the resolved-GSERF, we perform experiments on a challenging sample of 17*β*-estradiol that contains crowded and overlapped NMRs (Figure 2). 17*β*-estradiol is an important biological steroid estrogen that is related to cell maturation, differentiation, and transformation in the human body [37]. It is intuitive that 17*β*-estradiol is a small molecule, but it has a complicated molecular structure with proton groups in a similar chemical environment and extensive *J*-coupling systems [Figure 2a]. The 1D ^1^H NMR spectrum reveals the challenging case of spectral congestion for 17*β*-estradiol studies [Figure 2b], in which observed peaks are crowded and overlapped together, particularly in the spectral region between 1.00 ppm and 3.00 ppm, and this would inevitably hinder correct peak assignments for composition determination and structure analysis. Fortunately, the resolved-GSERF presents an alternative to analyzing this challenging molecule by selectively exciting the targeted proton and disassembling its coupling correlation network. Herein, we choose protons H_11*β*_ and H_12*α*_ as examples and perform related resolved-GSERF experiments to reveal their *J*-coupling correlation information. It is clear that the peaks of protons H_11*β*_ and H_12*α*_ are buried in the crowded spectral region between 1.00 ppm and 1.50 ppm in the 1D ^1^H NMR spectrum [Figure 2b], and it is impossible to selectively excite these two protons and extract their spectral information for analysis. In the resolved-GSERF experiment, we first target a resolved peak of proton H_11*α*_ and record its 1D selective TOCSY spectrum [Figure 2c]. Then we adopt an optimized short spin-lock mixing time of the DIPSI-2 to transfer the coupling correlation network of proton H_11*α*_ to protons H_11*β*_ and H_12*α*_ exactly, along with the preservation of satisfactory signal intensity. From the selective TOCSY spectrum in Figure 2c, it is seen that peaks from proton H_11*α*_ and its directly coupled protons H_9_, H_11*β*_, H_12*α*_, and H_12*β*_ are observed, and all these peaks are well-resolved and free of spectral congestion. This allows one to further extract *J*-coupling correlation information for protons H_11*β*_ and H_12*α*_ by the resolved-GSERF. In the subsequent resolved-GSERF experiment, proton H_11*β*_ is selectively excited by the slice-selection-based GSERF refocusing encoding scheme, and the resulting 2D resolved-GSERF spectrum can be recorded [Figure 2d]. In this 2D spectrum, the peak of proton H_11*β*_ is singlet, and its coupled protons H_9_, H_11*α*_, H_12*α*_, and H_12*β*_ give doublet peaks. It is ready to measure these doublet peaks for their *J*-coupling constants of coupled proton pairs from proton H_11*β*_, i.e., 1.87 Hz for the coupled proton pair of H_11*β*_ and H_9_, 13.49 Hz for H_11*α*_ and H_11*β*_, 12.93 Hz for H_11*β*_ and H_12*α*_, and 4.70 Hz for H_11*β*_ and H_12*β*_, respectively. Similarly, proton H_12α_ can also be targeted for the corresponding 2D resolved-GSERF spectrum [Figure 2e], in which three doublet peaks coupled to proton H_12*α*_ are observed and related *J*-coupling constants are measured as 4.43 Hz for proton H_12*α*_ H_11*α*_, 13.02 Hz for H_12*α*_ and H_11*β*_, and 13.15 Hz for H_12*α*_ and H_12*β*_, respectively. The comparison of *J*-coupling constants between protons H_11*β*_ and H_12*α*_ measured in two resolved-GSERF experiments [Figure 3d,e] reveals some discrepancies, and this error is still within an acceptable range. It is worth noting that the coupling constant 1.87 Hz is a small value. Benefiting from the high resolution of the resolved-GSERF spectrum, this small coupling constant can also be measured. After conducting a group of experiments on a complex sample of 17*β*-estradiol that contains crowded NMR resonances, we demonstrate that the resolved-GSERF method is a suitable approach for measuring *J*-coupling constants from spectral congestion, and its potential applications are self-evident.

We further demonstrate the applicability of the resolved-GSERF on a medical sample of amikacin to reveal targeted *J*-coupling correlation information. Amikacin belongs to a class of organic compounds and aminoglycoside antibiotics. Its mechanism of action involves acting on the bacterial ribosome, inhibiting protein synthesis, and compromising the integrity of the bacterial cell wall [38]. It has a relatively complicated molecular structure composed of aminoglycoside nucleotides and different residue groups, as shown in Figure 3a. Due to the complicated structure of amikacin, its resulting 1D ^1^H NMR spectrum suffers from spectral congestion, particularly in the 1.8–2.4 ppm and 3.3–4.2 ppm spectral regions [Figure 3b]. In these congested regions, observed peaks are presented in multiplets with extensive *J*-coupling splitting, and this gives rise to spectral crowding and makes spectral analysis of peak assignments and *J*-coupling determinations challenging. For instance, two targeted protons on CH*_β_* in the residue group, namely H*_βx_* and H*_βy_*, yield peaks crowded with other adjacent peaks in the 1D ^1^H NMR spectrum [Figure 3b]. The peak of proton H*_βx_* is overlapped with the peak of proton H_2*x*_, while the peak of proton H*_βy_* is crowded with the peak of proton H_2*y*_. To record these two peaks from the group of CH_2*β*_ in a high-resolution manner, we first perform a selective TOCSY scheme to selectively excite the peaks from the group of CH_γ_, and obtain a 1D selective TOCSY spectrum as shown in Figure 3c. Since the peak of protons H*_γ_* overlaps with that of proton H_6′*y*_, two groups of coupling correlation signals from both protons H*_γ_* and H_6′*y*_ are given in the 1D selective TOCSY spectrum. The selective *π* pulse in the selective magnetization transfer scheme of the resolved-GSERF experiment acts like a band selection and enables the simultaneous extraction of the coupling correlation network of proton H*_γ_* and H_6′*y*_. Benefiting from spectral simplification, peaks from the targeted protons H*_βx_* and H*_βy_* are isolated from the spectral congestion, and then the slice-selection-based GSERF refocusing encoding scheme in the resolved-GSERF experiment is further applied to target these two protons for the resulting 2D resolved-GSERF spectra [Figure 3d,e]. From the 2D resolved-GSERF spectrum targeting proton H*_βx_*, it shows that proton H*_βx_* is coupled to proton H*_βy_* with a *J*-coupling constant of 14.22 Hz, coupled to proton H*_α_* with a *J*-coupling constant of 7.55 Hz, and coupled to proton H*_γ_* with a *J*-coupling constant of 3.76 Hz [Figure 3d]. Similarly, the 2D resolved-GSERF spectrum targeting protons H*_βy_* reveals that the coupling correlation network from proton H*_βy_* to protons H*_βx_*, H*_γ_*, and H*_α_*, and their corresponding *J*-coupling constants are measured as 14.04 Hz, 7.55 Hz, and 9.41 Hz, respectively [Figure 3e]. The measured *J*-coupling constants from two resolved-GSERF spectra are consistent, which further validates the reliability of *J*-coupling measurements by the resolved-GSERF. Although the bandwidth of selective pulses adopted in the selective magnetization transfer scheme does not only cover the targeted peak, the resulting 2D resolved-GSERF spectra still present satisfactory information for analysis of the objective protons. Experiments on the complex sample of amikacin also verify the validity of the resolved-GSERF. It also demonstrates that the resolved-GSERF can select peaks in a specific region by band-selective pulses to extract the coupling network of targeted peaks, and subsequently give related *J*-coupling information.

## 3. Discussion

The resolved-GSERF presents a supplementary method to existing methodologies for analyzing accurate coupling networks of complex molecules. After exciting the given spectral peak by the selective magnetization transfer scheme, the targeted peak in the crowded and overlapped regions is extracted by the DIPSI-2 module with a proper spin-lock mixing time. Subsequently, the slice-selection-based GSERF refocusing encoding scheme is used to resolve the *J*-coupling information of these peaks. To validate the applicability of the resolved-GSERF, we have chosen different samples in the fields of biological metabolism and medical drugs. Experiments on strychnine, containing crowded NMRs in the 1D ^1^H spectrum, show the capability of resolved-GSERF to measure *J*-coupling constants from spectral congestion cases. A comparison experiment on a simple solution of *γ*-aminobutyric acid is also conducted between resolved-GSERF and conventional GSERF to show the measurement accuracy of the resolved-GSERF. Experiments on 17*β*-estradiol, a challenging molecule with crowded and overlapped peaks in 1D ^1^H NMR spectra, are performed to further illustrate the ability of the resolved-GSERF in restoring the *J*-coupling splitting pattern from the overlapping region. Moreover, even if none of the spectral peaks correlated to the congested targeted peak are isolated peaks in the 1D ^1^H spectrum, resolved-GSERF can also extract the coupling networks of the target peaks by using band-selective pulses and provide satisfactory *J*-coupling measurement results. This expands the application scope and has been experimentally validated by experiments on a medical sample of amikacin. The experimental results of resolved-GSERF on four groups of representative samples have shown its good performance in accurately measuring *J*-coupling information of crowded or even overlapped spectral peaks, which lays the groundwork for resolved-GSERF’s potential application in structure analysis of more complex practical samples.

Currently, the existing state-of-the-art methods for extracting *J*-coupling constants in crowded or overlapping spectral regions are the Clean G-SERF method and the TOCSY-GSERF method. In the Clean G-SERF experiment, selective COSY is used to extract peaks in crowded regions. However, it is theoretically known that selective COSY results in a loss of half the signal intensity before the mixing period. This significant sensitivity loss makes it impractical to reproduce the Clean G-SERF method on our 500 MHz NMR spectrometer. As for the TOCSY-GSERF method, the main difference from resolved-GSERF lies in the use of the PSYCHE module to achieve *J*-refocusing purposes, instead of the slice-selection module. PSYCHE and slice selection are two classical *J*-refocusing techniques widely used in the fields of homonuclear decoupling and selective refocusing. However, these two methods achieve *J*-refocusing purposes through different principles. For the PSYCHE module, *J*-refocusing principles are based on population selection. In contrast, the slice-selection module mainly relies on spatial encoding schemes to achieve *J*-refocusing. Moreover, benefiting from the characteristic of spatial encoding, the selective pulses in the slice-selection module can be replaced with multi-band pulses, and the sensitivity of the signal can be significantly improved using a Fourier phase encoding strategy. This potential is something that PSYCHE cannot possess. Another difference between PSYCHE and slice selection is that the PSYCHE scheme cannot be integrated with real-time acquisition, while the slice selection module can. In other words, the introduction of the resolved-GSERF method has laid a certain foundation for the rapid acquisition of *J*-coupling information for overlapping peaks. The above three merits have been proven effective in previously published spatial encoding NMR studies [26,34,35]. Furthermore, the TOCSY-GSERF method uses an echo/anti-echo scheme to provide pure absorption line shapes, improving spectral resolution through two experiments. However, for experiments like resolved-GSERF or TOCSY-GSERF, the number of peaks provided in the resulting spectra is already limited and, in most cases, sufficient for high resolution. Thus, sacrificing experimental convenience for absorption line shape may not always be the best choice. The core goal of the present Resolved-GSERF is not to demonstrate universal superiority over all existing NMR approaches, but to offer a practical workflow to extract accurate *J*-coupling constants from severely congested and overlapping proton spectra, which has been fully supported by the test results of strychnine, 17*β*-estradiol, amikacin and *γ*-aminobutyric acid samples. Based on the above discussion, we believe that although the existing state-of-the-art methods for providing high-resolution *J*-coupling information in crowded spectral regions share the same goal, the implementation and considerations differ. Therefore, we only conduct principle-level comparisons with representative state-of-the-art methods, rather than extensive quantitative performance comparisons. We chose GSERF as our comparative experiment because it is a classic application in the field of coupling constant measurements. We have validated the accuracy of the resolved-GSERF method through comparative experiments on a *γ*-aminobutyric acid sample whose 1D ^1^H spectrum had high-resolution characteristics and demonstrated the ability of resolved-GSERF to provide clean spectra under high sensitivity conditions in a strychnine sample. Therefore, we believe that our proposed resolved-GSERF method is novel and has the potential to advance this field in a unique way.

**Figure 4:**
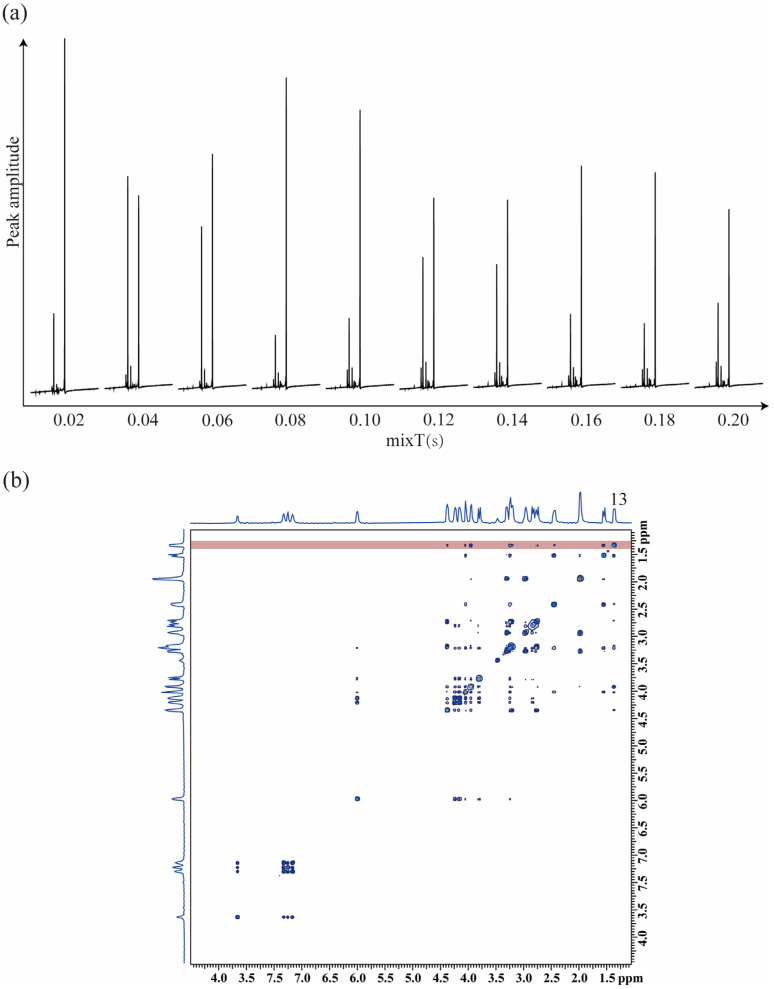
Verification experiment of transfer efficiency in the TOCSY experiment on strychnine: (**a**) A series of 1D selective TOCSY spectra as the mixT increases from 0.02 s to 0.20 s in 0.02 s increments. (**b**) 2D TOCSY experiment with a mixing time set to 0.20 s, highlighting the spectrum peaks that are totally correlated with proton Hc in the pink box.

To evaluate the transfer efficiency of TOCSY, we performed TOCSY experiments on a sample of strychnine (Figure 4). Firstly, in the selective TOCSY experiment, we selectively excited the peak of proton H_13_ and incremented the spin-lock mixing time (mixT) from 0.02 s to 0.20 s with a 0.02 s interval. The experimental results are shown as a series of 1D spectra [Figure 4a]. The peak intensity of proton H13 varied with the increase in mixT, attributed to the sinusoidal modulation related to mixT during the mixing period and the transfer of signal intensity to its coupled protons. Furthermore, as the mixing time was increased from 0.02 s to 0.08 s, more proton signals coupled to proton H_13_ were shown in the 1D spectrum. However, as the mixT was increased from 0.08 s to 0.20 s, the number of proton peaks coupled to H_13_ was no longer increased; only the intensities of the peaks were changed correspondingly with the increase in mixT. Subsequently, the 2D TOCSY experiment with the mixT of 0.20 s was performed on the sample of strychnine [Figure 4b], further validating the transfer efficiency of TOCSY. The obtained 2D TOCSY spectrum provided total correlation information of all the protons in the strychnine sample. Based on the experimental result, it was evident that despite the large mixT setting, the correlation of each proton was transferred within a limited number of protons, rather than all the protons throughout the entire molecule. These two experiments demonstrate the limited transfer efficiency of the spin-lock module in the magnetization transfer scheme. With the increase in mixT, the correlation can be transferred from three-bond coupling to long-range coupling. However, this correlation transfer is not unlimited. In fact, for some short-chain molecules, the coupling correlation can be generally transferred throughout the entire molecule. However, for longer molecular chains, the transfer of coupling correlation is limited to a finite number of protons. Additionally, to assess the impact of the transfer efficiency of the selective magnetization transfer scheme on the SNR, we conducted a comparison of the SNR between GSERF and resolved-GSERF experiments using a sample of *γ*-aminobutyric acid. Specific experimental results are provided in the Supplementary Materials.

Furthermore, the application of resolved-GSERF involves the use of multiple selective pulses, and here we explain the selectivity of selective pulses. The bandwidth of selective pulses is inversely proportional to their pulse width. In simple terms, a larger pulse width results in a smaller bandwidth and better selectivity. When using selective pulses to isolate specific peaks in the 1D ^1^H spectrum, the pulse selectivity depends on the difference in chemical shift between the specific peak and its nearest neighbors. In the slice selection scheme composed of selective pulses and weak encoding gradients, the weak encoding gradients uniformly encode the sample, allowing the selective pulses to act on different spins in different slices. The strength of the gradient determines the encoded frequency range ∆*υ* on the sample. Therefore, if frequency encoding is required for a sample of length *L*, the gradient strength is given by *G* = 2*πL*∆*ν*/*γ*.

It should be clarified that the current version of Resolved-GSERF has certain limitations. When strong couplings exist between the target proton and its adjacent coupled protons, highly selective pulses are mandatory, which inevitably sacrifices signal intensity and may prevent accurate *J*-coupling quantification due to poor sensitivity. Accordingly, Resolved-GSERF cannot deliver precise coupling constants for strongly coupled spin systems. Weak coupling dominates proton–proton interactions in most organic molecules, while strong coupling only appears as a special scenario, and dedicated pulse sequences for strong-coupling decoupling have long formed an independent research branch. The present work mainly targets weakly coupled systems, and strong-coupling analysis is not within the core scope of this study. To date, no existing NMR approach can extract reliable *J*-coupling values from congested overlapping peaks under strong coupling conditions, leaving this a valuable direction for our future investigation.

Additionally, the multiple selective pulses incorporated in the sequence introduce an inherent trade-off between spectral selectivity and signal sensitivity. The three unique strengths of slice-selection encoding—SNR improvement enabled by multiband pulses combined with Fourier phase encoding, intrinsic robustness against magnetic field inhomogeneity, and compatibility with real-time accelerated sampling—have been proven effective in previously published spatial encoding NMR studies and reflect the expandable potential of the proposed pulse architecture. Corresponding follow-up research, including the integration of multiband pulses, real-time sampling optimization, and sequence adaptation for strongly coupled systems, will be fully designed and experimentally validated in our subsequent independent work.

## 4. Materials and Methods

We selected various sample types, including complex chemical and biomedical samples, to systematically demonstrate the efficacy of resolved-GSERF in accurately measuring *J*-coupling constants of crowded spectral peaks. The first sample, strychnine (MedChemExpress, Shanghai Haoyuan ChemExpress Co., Ltd., Shanghai, China), was utilized to verify the feasibility of the resolved-GSERF method and to illustrate its implementation details. The second sample (Sigma-Aldrich, Sigma-Aldrich (Shanghai) Trading Co., Ltd., Shanghai, China), a challenging sample with a region of severe spectral peak overlapping in its 1D ^1^H spectrum, was employed to showcase the advantages of the resolved-GSERF method in extracting *J*-coupling information of congested spectral peaks. The third sample, amikacin (Amole, Shanghai Amole Biotechnology Co., Ltd., Shanghai, China), featured numerous congested spectral peaks on the ^1^D ^1^H spectrum. This sample demonstrated that resolved-GSERF could also obtain high-resolution *J*-coupling information even when the peaks correlated to the targeted peaks were not isolated, thus expanding its application range. Comparison experiments between resolved-GSERF and conventional GSERF were conducted on the *γ*-aminobutyric acid samples (Solarbio, Beijing Solarbio Science & Technology Co., Ltd., Beijing, China), confirming the accuracy of the *J*-coupling constants measured by resolved-GSERF. All the NMR experiments were conducted using a 500 MHz liquid-state NMR spectrometer (Varian, Agilent Technologies, Santa Clara, CA, USA) equipped with a 5 mm ^1^H/^13^C dual probe and a z-axis gradient with a maximum strength of 60 G/cm. Detailed experimental parameters were provided in the Supplementary Materials.

**Figure 5:**
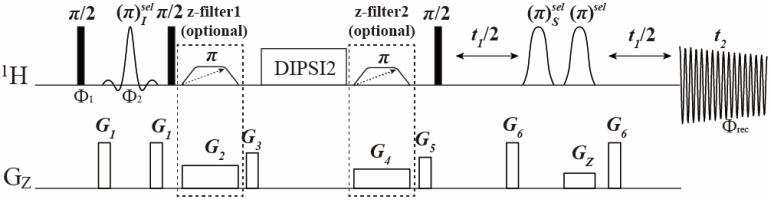
Pulse sequence diagram for resolved-GSERF experiments applicable to accurate *J*-coupling measurements on complex samples. The resolved-GSERF pulse sequence is composed of selective TOCSY and GSERF schemes. Black vertical bars stand for a *π*/2 hard pulse. Reburp and Gaussian waveforms stand for selective *π* pulses, respectively. Trapezoid-shaped pulses with arrows are frequency sweep pulses used in the z-filter element. *G*_1_, *G*_3_, *G*_5_, and *G*_6_ indicate coherence selection gradients. *G*_2_ and *G*_4_ are slice selection gradients for two *z*-filter elements, and *G_z_* is the slice selection gradient for the GSERF. The DIPSI2 is the composite pulse specifically designed for isotropic mixing purposes. Phase cycling is set as *Φ*_1_ = *x*, −*x*; *Φ*_2_ = *x*, *x*, *y*, *y*, −*x*, −*x*, −*y*, −*y*; *Φ_rec_* = *x*, −*x*, −*x*, *x*.

The designed pulse sequence diagram for the resolved-GSERF is shown in Figure 5. The whole pulse sequence is constructed by cascading the selective magnetization transfer module and slice-selection-based GSERF refocusing encoding module. These two schemes can achieve a satisfactory fusion for recording the desired spectral information, and they are independent of each other; thus, it is convenient for pulse sequence design and experimental setting. The selective magnetization transfer scheme employs three *π*/2 hard pulses, a selective *π* pulse with a pair of coherence selection gradient *G*_1_, a DIPSI-2 pulse element with two coherence selection gradients *G*_3_ and *G*_5_, and two optional *z*-filter elements with *π* chirp pulses matched by simultaneous gradients *G*_2_ and *G*_4_. This scheme is used to selectively excite a proton spin of a given proton and transfer its coupling correlation network between directly coupled protons and finally yields the resulting 1D spectrum that only contains signals coupled with the given proton. Optimized short DIPSI-2 spin-lock mixing only enables magnetization transfer between directly coupled protons to filter redundant off-target signals and simplify final spectra. The slice-selection-based GSERF refocusing encoding scheme contains a single selective *π* pulse, two equal evolution periods *t*_1_/2, a selective *π* pulse matched by a simultaneous weak gradient *G_z_*, and a pair of coherence selection gradients *G*_6_, and it is adopted after the selective magnetization transfer module to further extract *J*-coupling information of the targeted proton.

The resolved-GSERF sequence starts with a *π*/2 hard pulse to excite all proton spins, and the selective *π* pulse accompanied by a pair of coherent gradients *G*_1_ is adopted to selectively preserve the given proton spin while filtering out others. Subsequently, the core part of the selective magnetization transfer scheme, namely *π*/2–*z*-filter–*G*_3_–DIPSI2–*z*-filter–*G*_5_–*π*/2, would transfer the coupling correlation network from the given proton spin. In detail, the first *π*/2 hard pulse converts the transverse magnetization of the given proton spin into the longitudinal magnetization, and the first optional *z*-filter is adopted to eliminate residual signals and only retain the magnetization of the given proton spin along the *z*-direction. After that, the DIPSI-2 element takes effect on the desired magnetization along the z-direction and generates a strong *J*-coupling interaction among all spins. This strong *J*-coupling interaction propels the spins into a collective spin pattern, and then the *J*-coupling correlation effect is transferred from the given proton spin to other proton spins that are connected by chemical bonds. In general, the mixing time of DIPSI-2 determines the propagation distance of the *J*-coupling correlation network along the chemical bonds. A relatively long spin-lock mixing time in the DIPSI-2 can be set to obtain the whole coupling correlation network and yield isolated coupling signals of the given proton. Considering the negative effects of redundant spectral peaks, we generally adopt a shorter mixing time for the DIPSI-2 to transfer the *J*-coupling correlation effect among directly coupled proton spins, thus retaining high signal intensity and avoiding undesirable axial peaks and unwanted *J*-couplings. The second optional *z*-filter is further employed to eliminate residual signals from the desired magnetization transfer signals, and then the second *π*/2 hard pulse would convert the desired correlation signal term into observable signals. As for the *z*-filter module, it is an option in the resolved-GSERF to further filter out magnetizations other than that along the *z*-direction during the spin-lock module, thereby yielding cleaner spectra. However, the use of *z*-filter modules will prolong the total experimental time. In addition, as the duration of z-filter modules increases, the magnetizations along the -*z*-axis will scatter during the recovery to the *z*-axis, thereby reducing signal intensity. For experiments on complex samples with shorter relaxations, we can turn off the *z*-filter modules to ensure signal intensity. In this case, dephasing gradients *G*_3_ and *G*_5_ can also filter out unwanted magnetizations.

After the effect of the selective magnetization transfer scheme, the desired signal of a targeted proton that is coupled with the given proton can be extracted from an interferential signal background, such as spectral congestion with peak crowding or overlapping. The slice-selection-based GSERF refocusing encoding scheme is subsequently implemented to record *J*-couplings associated with the selected proton spin. It should be noticed that the targeted proton spin in the slice-selection-based GSERF refocusing encoding scheme can be different from the given proton spin in the selective magnetization transfer scheme. The given proton in the selective magnetization transfer scheme is chosen as a resolved peak away from the spectral congestion region to extract the desired magnetization transfer signals of the targeted proton. The targeted proton in the slice-selection-based GSERF refocusing encoding scheme can be chosen from the resulting 1D TOCSY spectra to obtain its *J*-coupling information. In the slice-selection-based GSERF refocusing encoding scheme, the first selective *π* pulse is performed on the targeted proton spin, and the second selective *π* pulse with a simultaneous weak gradient *G_z_* is performed to allow the *J*-coupling effect associated with the targeted proton spin to evolve during two equal evolution periods *t*_1_/2. The applied weak gradient spatially encodes the sample by correlating nuclear precession frequencies with spatial positions, so as to group different nuclei. Combined with selective pulses, this strategy enables effective grouped manipulation of targeted nuclear spins. After the *t_2_* acquisition period, Fourier transformation can be directly performed on the acquired resolved-GSERF data to obtain the resulting 2D spectrum, which records *J*-coupling information associated with the targeted proton along the *F*1 dimension and chemical shift information free of spectral congestion along the *F*2 dimension. Detailed theoretical derivation for signal evolution and related data processing of the resolved-GSERF are provided in the Supplementary Materials.

## 5. Conclusions

In conclusion, we introduce an efficient NMR method, resolved-GSERF, to achieve accurate *J*-coupling measurements on targeted protons from the spectral congestion, thus aiming for analyses on complex chemical and biological samples. The resolved-GSERF adopts the concept of targeted signal separation by the selective magnetization transfer scheme and accurate *J*-coupling determination by the slice-selection-based GSERF scheme. The combination of the selective magnetization transfer scheme and slice-selection-based GSERF refocusing encoding schemes facilitates accurate *J*-coupling measurements on peaks in crowded or even overlapped spectral regions. The performance of resolved-GSERF is demonstrated by experimental observations on a chemical sample of strychnine suffering from spectral congestion, a biological molecule of 17*β*-estradiol with crowded NMRs, and a medicine sample of amikacin with a complicated molecular structure. Benefiting from signal filtering on targeted *J*-coupling information, acceptable signal intensity, and convenient pulse sequence extension, the resolved-GSERF provides a robust supplement to existing NMR methods for characterizing *J*-coupling information of constant values and correlation networks in chemical and biological applications.

## Data Availability

The data that support the findings of this study are available from the corresponding author upon reasonable request.

## Supplementary Materials

The following supporting information can be downloaded at https://www.mdpi.com/article/10.3390/molecules31132386/s1, Figure S1: Pulse sequence diagram for GSERF; Figure S2: Comparison of experimental results of resolved-GSERF and conventional GSERF on γ-aminobutyric acid.

## Abbreviations

The following abbreviations are used in this manuscript:

SERF	Selective ReFocusing
TOCSY	Total Correlation SpectroscopY
NMR	Nuclear magnetic resonance
1D	One-dimensional
2D	Two-dimensional
SNR	Signal-to-noise ratio
