# Supplementary material for: Resolved-GSERF: A Proficient NMR Approach for Identifying Accurate J-Coupling Constants of Targeted Peaks from Spectral Congestion
Source: Molecules. 2026 Jul 7;31(13):2386. doi: 10.3390/molecules31132386 (PMC13362618; doi:10.3390/molecules31132386)
Supplement: Supplementary file 1 [file molecules-31-02386-s001.zip › molecules-4351524-supplementary.pdf]

Supplementary Material

Resolved-GSERF: A proficient NMR approach for identifying accurate *J*-coupling constants of targeted peaks from spectral congestion

Xiaoqing Lin

Zhangzhou institute of technology, Zhangzhou 363000, China

Table of Content

S1. Theoretical derivation for resolved-GSERF

S2. Experimental section

S3. Experimental results

S1. Theoretical derivation for resolved-GSERF

The pulse sequence of the resolved-GSERF is depicted in Figure 1 of the main text with a comprehensive description provided in the METHOD section. To intuitively comprehend the signal evolution of the resolved-GSERF, we examine a simple solution sample of an *AXM* spin-1/2 weakly coupled system. Here, *A*, *X*, and *M* represent the spin *I*, *S*, and *S*' respectively. There exists a coupling relationship between spin *I* and *S* with a coupling constant of J_{IS}, and between spin *S* and *S*' with a coupling constant of $J_{SS'}$. However, there is no direct coupling relationship between spin *I* and *S*. We then derive the theoretical expression of the resulting resolved-GSERF signals. It is assumed that Ω_I and Ω_S are frequency offsets of the spins *I* and *S* in the rotating frame. In the subsequent deduction, we simplify the derivation procedure by omitting the effects of radiation damping, diffusion, and intermolecular nuclear Overhauser effect. The evolution of magnetization is elucidated using the reduced density operator. For the *AXM* spin-1/2 coupled system under discussion, the reduced density operator at the initial thermal equilibrium state with high-temperature approximation can be represented as follows:

$$\sigma_0 = I_z \tag{S1}$$

In the selective TOCSY of resolved-TOCSY, the coherent selection path of spin *I* follows the sequence: 0→±1→∓1→0→+1; In the GSERF of resolved-TOCSY, the coherent selection path of spin *S* is +1→-1. Under the influence of non-selective *π*/2 pulses, terms I^+ and I^- are generated. Subsequently, a selective *π* pulse, paired with coherent selection gradients, scatters the spins of all protons, excluding spin *I*. This results in the following conditions:

$$\sigma_0 \xrightarrow{\pi/2I_x} -\frac{1}{2i}\left(I^+ - I^-\right) \xrightarrow{\pi I_x} -\frac{1}{2i}\left(I^- - I^+\right) \tag{S2}$$

Following this, a mixing period of selective TOCSY is initiated, during which a non-selective pulse of *π*/2 is applied at the first. This application results in the production of terms I^+, I^- and I_z. Subsequently, the implementation of the z-filter module or dephasing gradient enables the filtration of all terms excluding I_z. This process is represented as follows:

$$-\frac{1}{2i}\left(I^{-}-I^{+}\right)\xrightarrow{\pi/2I_x}\xrightarrow{z-filter}-I_z \tag{S3}$$

Under the influence of spin-lock module, the spins exhibit a strong coupling relationship, eliminating individual spin modes and leading them to process in a collective spin pattern. In this context, the duration of spin-lock module is set to be short, ensuring that coupling action is transferred only among the directly coupled protons, similar to the action observed in conventional selective COSY. Subsequently, another z-filter or dephasing gradient is employed to filter out terms other than I_z, thereby maintaining the cleanliness of the signal. This process is as follows:

$$-I_z\xrightarrow{spin-lock}\xrightarrow{z-filter}-I_z\cos^2\left(\pi J_{IS}\tau\right)-S_z\sin^2\left(\pi J_{IS}\tau\right) \tag{S4}$$

At the end of the selective TOCSY, another non-selective $\pi/2$ is applied to generate +1 order term for the next step. Since the frequency center of the selective pulse in the GSERF is aligned with the spin S, the subsequent derivation process only considers the spin S:

$$-S_z\sin^2\left(\pi J_{IS}\tau\right)\xrightarrow{\pi/2}-\frac{1}{2}S^{+}\sin^2\left(\pi J_{IS}\tau\right) \tag{S5}$$

During the first $t_1/2$ evolution period in GSERF, the spin S undergoes the following evolution:

$$\begin{aligned}&-\frac{1}{2}S^{+}\sin^2\left(\pi J_{IS}\tau\right)\xrightarrow{t_1/2}\\&\sigma_1=-\frac{1}{2}S^{+}\sin^2\left(\pi J_{IS}\tau\right)e^{-i\Omega_S\frac{t_1}{2}}\left[\cos\left(\pi J_{SS'}\frac{t_1}{2}\right)-i2S_z\sin\left(\pi J_{SS'}\frac{t_1}{2}\right)\right]\end{aligned} \tag{S6}$$

Then, a selective π pulse and a selective π pulse with a slice selection gradient are applied, their frequency centers are both on the spin S. A pair of coherent selection gradients on either side of the two selective π pulses filter out the spin S signal. Since the slice selection gradient spatially frequency encodes the sample, the signal of spin S' evolves as follows after another $t_1/2$ evolution period:

$$\begin{aligned}&\sigma_1\xrightarrow{(\pi)_S}\xrightarrow{(\pi)_{S'}}\xrightarrow{t_1/2}\\&-\frac{1}{2}S^{-}\sin^2\left(\pi J_{IS}\tau\right)\left[\cos\left(\pi J_{SS'}t_1\right)+i2S_z{}'\sin\left(\pi J_{SS'}t_1\right)\right]\end{aligned} \tag{S7}$$

Finally, during the t_2 sampling period, we can acquire observable signals as

$$-\frac{1}{2}S^{-}\sin^{2}\left(\pi J_{IS}\tau\right)\left[\cos\left(\pi J_{SS'}t_{1}\right)+i2S_{z}'\sin\left(\pi J_{SS'}t_{1}\right)\right]\xrightarrow{t_2}$$
$$\frac{1}{2}S^{-}e^{i\Omega_{S}t_{2}}\sin^{2}\left(\pi J_{IS}\tau\right)\cos\left[\pi J_{SS'}\left(t_{1}+t_{2}\right)\right] \tag{S8}$$

S2. Experimental section

All experiments were conducted using a 500 MHz liquid nuclear magnetic resonance spectrometer (Varian, Agilent Technologies, Santa Clara, CA, USA), with a consistent temperature of 298 K maintained throughout the process. The conventional GSERF pulse sequence was shown in Figure S1, and the selective pulses used were all in Gauss shape. In the resolved-GSERF experiment, the selective π pulses in the selective TOCSY scheme adopted the Reburp shape, while the Gaussian shape in the GSERF scheme. The z-filter module was activated in all individual selective TOCSY experiments.

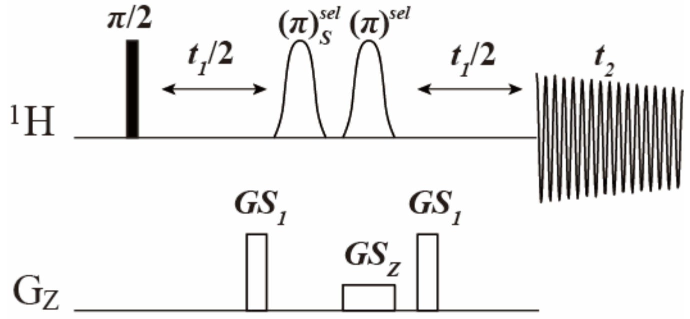

Figure S1. Pulse sequence diagram for GSERF. Black vertical bars and Gaussian waveforms stand for $\pi/2$ hard pulse and selective π pulse, respectively. GS_1 indicates coherence selection gradients. GS_z is the slice selection gradient for the GSERF.

Firstly, a 200 mM solution of strychnine dissolved in chloroform-*d* was employed to verify the feasibility of the resolved-GSERF method. The experimental parameters of the resolved-GSERF sequence, which include the duration of the non-selective $\pi/2$ pulse (*T1*), the duration of the selective π pulse (*T2*), the gradient delay of the coherent selection gradient (*gt1* - *gt4*), the intensity of the coherent selection gradient (*g1* - *g5*), the duration of DIPSI-2 element (*mixT*), and the duration of the non-selective $\pi/2$ pulse in DIPSI-2 element (T_m) were set as follows: *T1*=10.85 μs, *T2*=54.167ms, *gt1*=1ms, *gt2*=1ms, *gt3*=1ms, *gt*=500μs, *g1*=22.03G/cm, *g2*=12.02G/cm, *g3*=16.02G/cm,

g4=46.88G/cm, *g5*=2.73G/cm, T_m=32.062μs, *mixT*=50ms. The z-filter module was disabled for these experiments. The spectral width of the direct sampling dimension (*SW*) and indirect sampling dimension (*SW1*) were set to 8000Hz and 40Hz, including 4800 increments and 20 increments. Recovery time (*d1*), transient scan count (*nt*), and virtual scan count (*ss*) were set as *d1*=1.5s, *nt*=32, and *ss*=8. The spectral center of the selective TOCSY aligned with the peak of proton H_{17}, while that of the GSERF aligned with the peak of proton $H_{18\alpha}$. The total duration of the experiment was about 29min. The parameters used by the GSERF were the same as those used in the GSERF of the corresponding resolved-GSERF. The total experimental time was about 28 min.

Secondly, a challenging sample, 200 mM estradiol dissolved in DMSO-*d* was also used to validate the feasibility of the method in the region of spectral peak overlap. The parameters were the same as those used by strychnine, except *T1*=10.95μs, *T2*=24.375ms, and *mixT*=30ms. In the two sets of the resolved-GSERF experiments, the spectral center of the selective TOCSY aligned with peak $H_{11\alpha}$. The spectral center of the GSERF aligned with the peak of proton $H_{11\beta}$ in the first set of the resolved-GSERF experiment, while it aligned with the peak of proton $H_{12\alpha}$ in the second set of the resolved-GSERF.

Thirdly, 200 mM amikacin dissolved in D_2O was used as a complex sample to validate the advantages of the resolved-GSERF method. The parameters were the same as those used by strychnine, except *T1*=10.95μs, and *mixT*=30ms. In the first set of resolved-GSERF experiments, the spectral center of the selective TOCSY aligned with the peak of proton H_γ, and that of the GSERF aligned with the peak of proton $H_{\beta x}$. In the second set of resolved-GSERF experiments, the spectral center of the selective TOCSY aligned with the peak of proton H_γ, and that of the GSERF aligned with the peak of proton $H_{\beta y}$.

In addition, a set of experiments on *γ*-aminobutyric acid using D_2O as the solvent were also performed to compare the accuracy of resolved-GSERF and conventional GSERF results. The parameters were set as: *T1*=11.1μs, *T2*=18.5ms, *gt1*=1ms, *gt2*=1ms, *gt3*=1ms, *gt4*=1ms, *g1*=22.03G/cm, *g2*=12.02G/cm, *g3*=16.02G/cm,

g4=46.88G/cm, *g5*=1.56G/cm, *mixT*=50ms, T_m=32.062μs. *SW*=8000Hz, *SW1*=60Hz, including 8000 increments and 40 increments, *d1*=1s, *nt*=1, *ss*=2. The z-filter module was enabled with specific parameters: the intensity of gradient used in the z-filter module *gz1*=9.42G/cm and *gz2*=11.31 G/cm, the duration of sweep pulse *Tz1*=30ms and *Tz2*=40ms. In the first set of the resolved-GSERF experiment, the spectral center of the selective TOCSY aligned with the peak of proton H_3, and that of GSERF aligned with the peak of proton H_1. In the second set of the resolved-GSERF experiment, the spectral center of the selective TOCSY aligned with the peak of proton H_3, and that of GSERF aligned with the peak of proton H_2. In the third set of the resolved-GSERF experiment, the spectral center of the selective TOCSY aligned with the peak of proton H_1, and that of the GSERF aligned with the peak of proton H_3. The total duration of the experiment was about 2 min. The parameters used by the GSERF were the same as those used in the GSERF of the corresponding resolved-GSERF. The total experimental time was about 2 min.

S3. Experimental results

To evaluate the accuracy of the coupling constant measured in the resolved-GSERF experiment, *γ*-aminobutyric acid ($C_4H_9NO_2$), a simple sample with high spectral peak resolution, is chosen for comparative experiments. *γ*-aminobutyric acid, a non-protein amino acid, is predominantly produced by plants, animals, and microorganisms. As a small molecular weight, *γ*-aminobutyric acid is safe for consumption and can be utilized in the manufacturing of beverages and various food products. Its structural diagram is depicted in Figure S2(a), with corresponding positions labeled with serial numbers for spectral peak attribution. In the 1D ^{1}H spectrum [Figure S2(b)], *γ*-aminobutyric acid exhibits three distinct spectral peaks with high resolution. The absence of overlapping among these multiplets facilitates the precise measurement of the coupling constant. To assign the spectral peaks in the 1D ^{1}H spectrum, the three peaks are labeled with serial numbers corresponding to those in the structure diagram. From the molecular structure of *γ*-aminobutyric acid [Figure S2(a)], it can be seen that there is a coupling relationship between proton 1 and 3 in its molecular structure, as well as between proton 2 and 3. Therefore, the spectral peaks of proton 1 and 2 are

triplets in the 1D ^1H spectrum, and accurate coupling constants can be obtained. The coupling constant of proton 1 ($J_{H1, H3}$) is measured to be 7.63 Hz, while the coupling constant of proton 2 ($J_{H2, H3}$) is measured to be 7.42 Hz. The conventional GSERF method, recognized for its accuracy in measuring the *J* coupling constants of spectral peaks, is initially employed to determine the accurate *J* coupling constants of each spectral peak. In the conventional GSERF experiment with the selective excitation of the peak from proton H_1 [Figure S2(c)], a pair of peaks appear on the indirect dimension at the chemical shift position of the peak of proton H_3 (1.84ppm), reflecting the coupling constant between protons H_1 and H_3. The half of distance between the two peaks is 7.53 Hz, which is the value of the coupling constant $J_{H1,H3}$. When the peak of proton H_2 is selectively excited in the conventional GSERF experiment [Figure S2(e)], the coupling constant between proton H_2 and its coupled proton H_3 can be obtained as 7.43 Hz. In the conventional GSERF experiment where the peak of the proton H_3 is selectively excited [Figure S2(g)], two pairs of peaks are found in the indirect dimension, which corresponds to the chemical shift positions of the protons H_1 and H_2, respectively. The half of distance between a pair of peaks at the chemical shift position of proton H_1 represents the coupling constant value between proton H_1 and H_3 ($J_{H1, H3}$), which is 7.66 Hz. This value is near the value of $J_{H1, H3}$ measured in the conventional 1D ^1H spectrum and GSERF with the selective excitation of the peak of proton H_1, and the errors are within an acceptable range. The half of distance between a pair of peaks at the peak chemical shift position of proton H_2 represents the coupling constant value between protons H_2 and H_3. The measured value of 7.38 Hz is similar to the $J_{H2,H3}$ values measured in the conventional 1D ^1H spectrum and GSERF experiment with the selective excitation of the peak of proton H_2. The measured parameters are compared to verify the accuracy of the new method resolved-GSERF.

In the resolved-GSERF experiment, we conducted three sets of comparative experiments. Since γ-aminobutyric acid is a small molecule, there is no need to worry about the loss of the strength of the targeted signal due to the signal transmission via coupling transfer. Therefore, the spin-locking duration is set to be longer

(*mixT*=50ms), and the coupling effect is transferred to the whole spin system. The resulting selective TOCSY spectrum shows three spectral peaks. In the first set of resolved-GSERF experiments, the spectral center of the selective TOCSY aligns with the peak of proton H_3, and that of GSERF aligns with the peak of proton H_1. Due to the coupling relationship between protons H_1 and H_3, a pair of peaks eventually appears in the indirect dimension of the chemical shift position of the peak of proton H_3. The value of $J_{H1,H3}$ measured by the resolved-GSERF is 7.68 Hz, which is similar to the result of $J_{H1,H3}$ measured by conventional GSERF [Figure S2(c)], and the error is within the acceptable range. In addition, compared with the spectral representation of resolved-GSERF and conventional GSERF, they are similar. In the second set of resolved-GSERF experiments, the spectral center of the selective TOCSY aligns with the peak of proton H_3, and that of GSERF aligns with the peak of proton H_2. The value of $J_{H2,\ H3}$ measured by resolved-GSERF is similar to that measured by conventional GSERF. In the third set of resolved-GSERF experiments, the spectral center of the selective TOCSY aligns with the peak of proton H_1, and that of GSERF aligns with the peak of proton H_3. Finally, the $J_{H1,H3}$ and $J_{H2,H3}$ obtained in the resolved-GSERF experiment are 7.62 Hz and 7.42 Hz, respectively, which meet the expectation. This comparative experiment not only validates the reliability of the results obtained from GSERF and resolved-GSERF experiments but also underscores the significance of employing GSERF as a comparative method.

To verify the impact of the transfer efficiency of the selective TOCSY module on the signal-to-noise ratio (SNR), we perform the SNR comparison between conventional GSERF and resolved-GSERF experiments on a sample of γ-aminobutyric acid. In both experiments, the experimental parameters in conventional GSERF were set the same as those in the GSERF module of the resolved-GSERF. Our measurements and calculations show that the SNR for GSERF is 196.4, while the SNR for resolved-GSERF is 75.9, indicating that the SNR loss is caused by the transfer efficiency of the TOCSY module. However, this loss in SNR is generally acceptable because when measuring coupling information from particularly crowded or overlapping spectral peaks, the SNR loss associated with the selective

pulses in conventional GSERF far exceeds the SNR loss in the TOCSY module of the resolved-GSERF. Furthermore, compared to the conventional GSERF method, resolved-GSERF provides cleaner, high-resolution spectra.

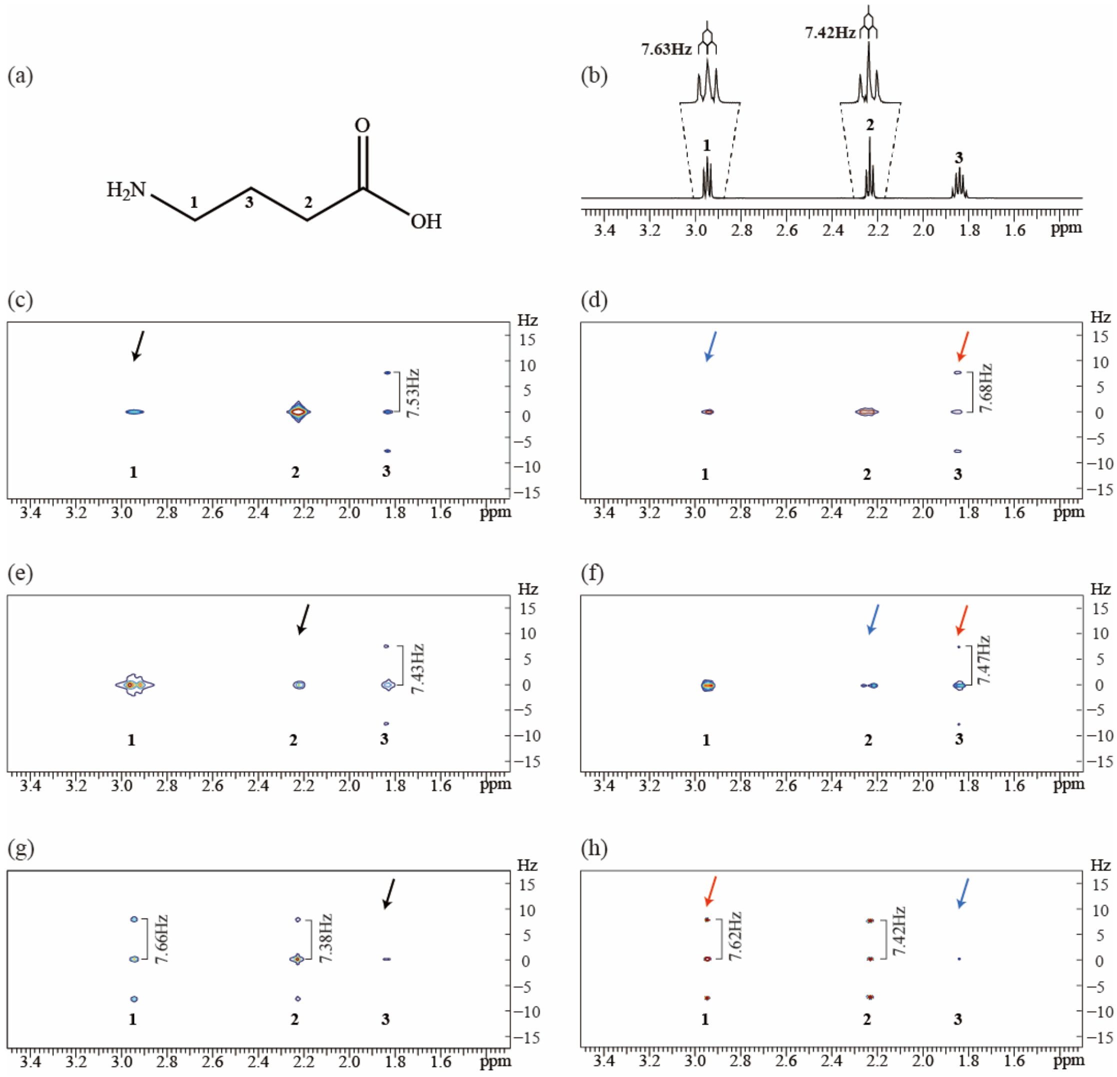

Figure S2. Comparison of experimental results of resolved-GSERF and conventional GSERF on γ-aminobutyric acid. (a) structure diagram of γ-aminobutyric acid. (b) 1D ^{1}H spectrum and the enlarged section shows the coupling constants of proton 1 and proton 2. GSERF experiments with the spectral center aligned with the peak of (c) proton H_1, (e) proton H_2, and (g) proton H_3. Resolved-GSERF experiments with the spectral center aligned with (d) the peak of proton H_3 in selective TOCSY and H_1 in GSERF, (f) the peak of proton H_3 in selective TOCSY and H_2 in GSERF, (h) the peak of proton H_1 in selective TOCSY and H_3 in GSERF.
